# Structure, Function, and Biology of the *Enterococcus faecalis* Cytolysin 

**DOI:** 10.3390/toxins5050895

**Published:** 2013-04-27

**Authors:** Daria Van Tyne, Melissa J. Martin, Michael S. Gilmore

**Affiliations:** Department of Ophthalmology and Department of Microbiology and Immunobiology, Harvard Medical School, Massachusetts Eye and Ear Infirmary, 243 Charles St., Boston, MA 02114, USA

**Keywords:** cytolysin, lantibiotic, bacteriocin

## Abstract

*Enterococcus faecalis* is a Gram-positive commensal member of the gut microbiota of a wide range of organisms. With the advent of antibiotic therapy, it has emerged as a multidrug resistant, hospital-acquired pathogen. Highly virulent strains of *E. faecalis* express a pore-forming exotoxin, called cytolysin, which lyses both bacterial and eukaryotic cells in response to quorum signals. Originally described in the 1930s, the cytolysin is a member of a large class of lanthionine-containing bacteriocins produced by Gram-positive bacteria. While the cytolysin shares some core features with other lantibiotics, it possesses unique characteristics as well. The current understanding of cytolysin biosynthesis, structure/function relationships, and contribution to the biology of *E. faecalis* are reviewed, and opportunities for using emerging technologies to advance this understanding are discussed.

## 1. Introduction: The Enterococci as Emergent Hospital Pathogens

Enterococci are ancient members of the animal microbiome that are believed to date back at least to the last common ancestor of mammals, reptiles, birds and insects in the early Devonian period, 412 million years ago [1]. These bacteria thrive in the nutrient-rich, oxygen-depleted environment of the intestinal tract, and at least in part because of shedding from animal hosts, are readily found in the environment [2]. They are core members of the commensal intestinal microbiota, which is densely colonized with up to 10^11^ bacterial cells/gram feces [3,4]. Enterococci are the predominant Gram-positive cocci found within this niche, and in humans they can be isolated at concentrations of 10^5^ to 10^7^ CFU/gram feces [5,6]. Bacteriophage induction in response to environmental cues is emerging as one strategy for enterococcal colonization and control in the intestinal ecosystem [7]. The presence of enterococci in the complex ecology of the gastrointestinal tract provides an ample reservoir where genetic exchange and selection can occur.

Enteroccoci are low-GC, Gram-positive, non-sporulating, facultative anaerobes that currently rank among the most prevalent multidrug resistant hospital pathogens worldwide [1]. They are the third most commonly isolated healthcare pathogen [8], and are capable of causing a variety of infections including endocarditis, sepsis, surgical wound infections, and urinary tract infections [5,9,10]. The genus Enterococcus consists of over 40 ecologically diverse species [5,11], yet more than 90 percent of enterococcal infections are caused by two species: *E. faecalis* and *E. faecium* [8,10,12]. The genomes of multidrug resistant enterococci consist of more than 25 percent mobile elements, which reflect a rampant accumulation of drug resistance elements and virulence factors [13]. Many enterococcal mobile elements are transferable by conjugation on pheromone-responsive plasmids, broad host range conjugative plasmids, or conjugative transposons [14,15]. The production of sex pheromone peptides by plasmid-free strains allows conjugative pheromone-responsive plasmids to transfer at rates as high as 10^−3^ to 10^−1^ per donor cell [16], efficiently disseminating virulence and antibiotic resistance genes between strains [17,18]. 

The horizontal transfer of mobile elements has contributed much to the evolving fitness of enterococci in hospital settings [11,18]. Since the 1960’s, hospital-associated enterococcal infections have become increasingly antibiotic resistant [19]. Antibiotic treatment results in a loss of protection from host colonization as well as reduced microbial species diversity among the intestinal microbiota. This provides an opportunity for drug resistant enterococci to invade the intestinal niche and proliferate uncontrollably [3,20]. Horizontally acquired antimicrobial resistances were first described in the 1970’s [21]. Analysis of an outbreak of multidrug resistant enterococcal bacteremia in the mid-1980s determined that half of all isolates were from the same hemolytic clone [22]. Subsequently, the first vancomycin-resistant clinical isolate of *E. faecalis*, strain V583, was isolated in the United States from the bloodstream of an infected patient [23]. Hospital endemic and epidemic multidrug resistant enterococcal infection rates have since continued to increase worldwide [24,25,26].

Enterococcal disease was first described in detail in the late 19th century, when an abundant Gram-positive diploccocus was isolated from patients with intestinal diseases that was similar to an organism isolated from healthy patients [27]. This saprophytic microbe, named ‘Enterocoque’, was initially difficult to culture, most likely due to now-appreciated nutrient auxotrophies [27,28]. Pathogenicity was reproduced in rabbit and mouse models, in which inoculation lead to severe infection and fatality [27]. A further report from Thiercelin [29] described translocation of the bacteria from the gastrointestinal tract to the bloodstream, resulting in septicemia. At about the same time, MacCallum and Hastings described a death due to enterococcal infection causing acute endocarditis [30]. Originally designated as *Micrococcus zymogenes*, the bacterium isolated from the blood and cardiac vegetations of the patient was used to intraperitoneally infected mice, rabbits and dogs, and was found to recapitulate the same endocarditis symptoms [30], satisfying Koch’s Postulates and establishing *Enterococcus* as the cause of the patient’s death.

The observation that some *E. faecalis* strains produced zones of hemolysis on blood agar plates led to the first comprehensive study of the hemolysin molecule [31]. Subsequently, hemolysis was found to be caused by a unique toxin, now termed cytolysin, as it lyses a broad range of target cells including both Gram-positive bacteria and eukaryotic cells [31,32,33,34,35]. The cytolysin is now known to make a large contribution to the pathogenicity of *E. faecalis* [36,37].

## 2. Cytolysin and Toxicity of Enterococcal Infections

The cytolysin toxin of *E. faecalis*, termed a “streptolysin” since it was produced by Lancefield group D *Streptococcus* and caused a zone of hemolysis on blood agar, was first experimentally characterized in 1934 [31]. *E. faecalis* (then called *Streptococcus faecalis*) was first considered to be pseudo-hemolytic, as hemolytic activity could rarely be detected in liquid broth but was readily seen on blood agar [31]. A gradient of erythrocyte susceptibilities, depending on species of origin, was observed, with human, horse, dog, rabbit and mouse erythrocytes being susceptible, and sheep and goose erythrocytes being resistant to lysis. A horse flesh infusion was derived that supported production of cytolysin in liquid culture, allowing for its characterization as a heat-labile, oxygen stable molecule (in contrast to the family of thiol-activated, cholesterol dependent cytolysins produced by other Gram-positive bacteria) [31]. In addition, hemolysin-producing enterococcal strains were observed to have bacteriocin activity against streptococcal strains and other Gram-positive bacteria [38,39,40]. The bactericidal and hemolytic phenotypes were experimentally characterized to be due to a single molecule. Brock *et al*. [39] showed that the hemolytic and bactericidal activities were both lost after UV irradiation and that restoration of one activity reestablished the other. The molecule was termed *E. faecalis* cytolysin to reflect the dual bactericidal and cytolytic activities exhibited [41]. 

The association of cytolysin expression and increased toxicity of enterococcal infections has been studied in multiple animal models, as well as in clinical outcomes (Table 1). Ike and Clewell first described enhanced virulence due to cytolysin expression in the mouse through dose-dependent intraperitoneal injection with *E. faecalis* strains harboring the plasmid pAD1, which encodes cytolysin [42]. After 7 days of infection with cytolysin negative strains (3 × 10^9^ CFU) all mice survived, while mice injected with cytolysin positive strains (≥10^9^ CFU) died within 4–5 h [43]. Subsequently, cytolysin positive variants were shown to lyse mouse erythrocytes, macrophages and polymorphonuclear neutrophils [44]. Toxicity due to cytolysin was also determined in a rabbit endocarditis model, whereby cytolysin and aggregation substance positive strains were lethal in 55 percent of infections, versus 15 percent in animals infected with only aggregation substance positive strains [45]. In rabbit endophthalmitis, cytolytic strains readily destroyed organ function and were untreatable, compared to isogenic, non-cytolytic strains [46,47]. When *C. elegans* is fed on lawns of cytolysin positive *E. faecalis*, death occurs faster than when fed on isogenic non-cytolytic bacteria [48]. 

**Table 1:** Contribution of the *E. faecalis* cytolysin to virulence.

Setting	Effect of Cytolysin	Reference
**Human bacteremia**	Cytolysin makes infection five times more acutely lethal	[22]
**Rabbit endophthalmitis**	Cytolysin makes infection acutely destructive to retina and other ocular structures, and refractory to antibiotic treatment	[46,47,49]
**Mouse intraperitoneal infection**	Cytolysin makes infection approximately one hundred times more acutely lethal	[42,50]
**Rabbit endocarditis**	Cytolysin makes infection acutely lethal in synergy with aggregation substance	[45]
***C. elegans* ingestion**	Cytolysin makes infection acutely lethal following ingestion	[48]

The cytolysin has also been shown to be associated with increased toxicity in human infection. A retrospective study analyzed 190 clinical *E. faecalis* isolates and found that 45 percent of isolates were cytolysin positive. Furthermore, even after controlling for treatment modality and drug resistance, patients infected with cytolytic *E. faecalis* were at a five-fold increased risk of an acutely terminal outcome (death within three weeks of diagnosis) compared to patients infected with non-cytolytic strains [22]. *E. faecalis* can cause a severe postoperative endophthalmitis, and cytolytic strains have been found to be common in these infections [51]. Epidemiological studies from Japan found that 60 percent of *E. faecalis* isolates analyzed from two hospitals were cytolysin positive [52]. Another study found that hemolysis was common to all clinical enterococci isolates investigated (which is not typical), while only six percent of food isolates were hemolytic [53]. In addition to causing increased toxicity of infection, the bacteriocin activity of the cytolysin may well be an important colonization factor of *E. faecalis* in the intestine, prior to establishment of infection at another sterile body site. *In vitro* experiments showed that cytolytic strains can outcompete bacteriocin-sensitive enterococci and other Gram-positive bacteria in liquid broth culture [39]. Cytolysin was also observed to be produced by *E. faecalis* isolated from nine out of 31 healthy infants in Norway [54].

Although a broad understanding of the genetics and biosynthesis of cytolysin is fairly advanced, many of the details of its production, as well as the precise mechanism by which it contributes to the pathogenesis of infection, are not well known. Hypothetically, the ability to lyse intestinal epithelial cells may allow *E. faecalis* to access the blood stream in order to travel to and colonize distant sites, such as the heart valve. Additionally, the ability to lyse mouse neutrophils and macrophages might contribute to immune evasion [44]. Other *E. faecalis* products, such as gelatinase and capsular polysaccharides, have been shown to help the bacteria to circumvent host immunity [55,56], but the precise role that the cytolysin might play in immune evasion is still unknown.

## 3. Cytolysin Structure and Function

### 3.1. Overview of the Cytolysin

**Figure 1:**
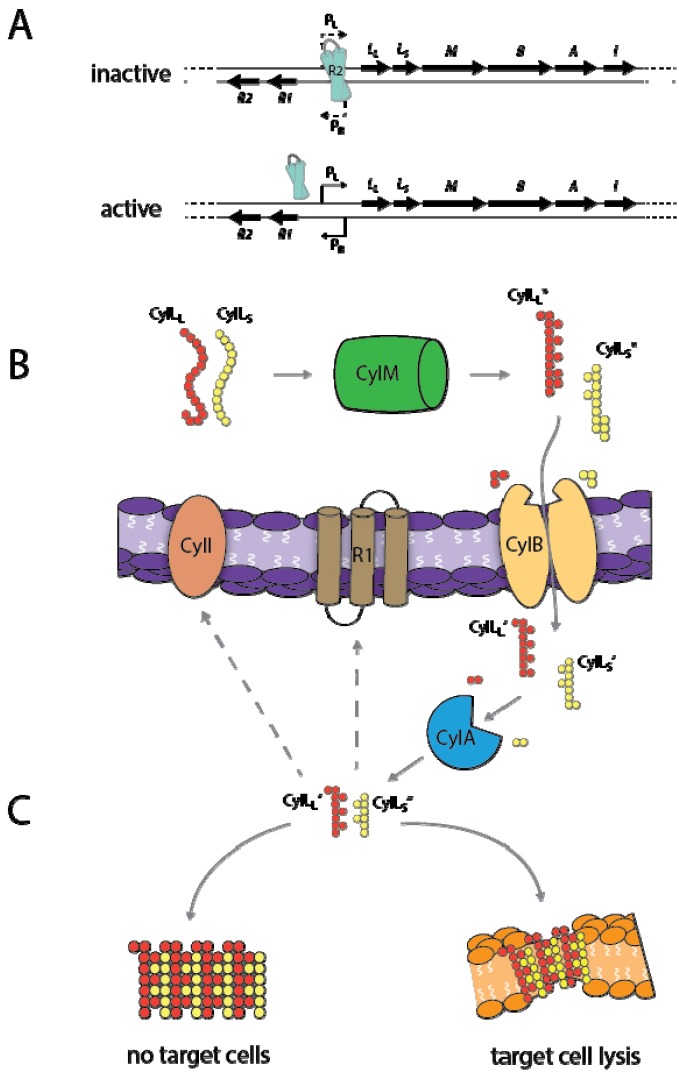
***E. faecalis* cytolysin expression.** (**A**) Cytolysin operon in the inactive and active states. In the inactive state, CylR2 binds to the P_Lys_ (P_L_) promoter [68]. Autoinduction via quorum sensing triggers an inferred change in the binding of the cytolysin promoter by the CylR2 protein, resulting in high-level expression of the cytolysin operon [67]. (**B**) Cytolysin processing and secretion. Large and small subunits are post-translationally modified by CylM [65], secreted and trimmed by CylB [41], and further processed by CylA [64]. (**C**) Cytolysin activity, in the absence and presence of target cells. In the absence of target cells the subunits form inactive and insoluble multimeric complexes. In the presence of target cells they coordinate to form a pore in the target cell membrane [71].

A general scheme for cytolysin production, processing, secretion, and regulation is shown in Figure 1. Enterococci produce a wide array of bacterocins, but the cytolysin is the only well characterized lantibiotic produced by *E. faecalis* [57]. Cytolysin production is a variable trait among *E. faecalis* isolates [16,52,58]. Among cytolysin-producing strains, the operon is either chromosomally-encoded within a 150-kilobase pathogenicity island (PAI) [43,59,60], or on a conjugative, pheromone-responsive plasmid, such as pAD1 [61,62,63]. The cytolysin operon consists of six genes related to toxin biosynthesis, as well as two divergently transcribed genes encoding regulatory proteins [41,64,65,66,67] (Figure 1A). In the inactive state, the cytolysin repressor protein CylR2 binds to the P_Lys_ (P_L_) promoter [68]. Low-level transcription of the operon is believed to result in basal production of a small amount of the cytolysin subunits [67]. Autoinduction via quorum sensing in the presence of target cells triggers an inferred change in the binding of the cytolysin promoter by the CylR2 protein, resulting in high-level expression of the cytolysin operon [67]. 

The functional cytolysin toxin consists of large and small subunit oligopeptides, encoded by the genes *cylL_L_* and *cylL_S_*, respectively [64] (Figure 1B). CylL_L_ and CylL_S_ primary translation products undergo extensive post-translational modification, including dehydration of serine and threonine residues, and subsequent formation of intramolecular lanthionine and methyllanthionine bridges between these dehydrated residues and nearby cysteine thiol groups within each subunit [69,70]. Dehydration of the toxin subunits in the initial modification step is inferred to be catalyzed by the CylM protein [65]. The ATP-binding cassette (ABC) transporter CylB secretes and trims the CylM-modified peptides CylL_L_^*^ and CylL_S_^*^ [41], resulting in externalization of CylL_L_’ and CylL_S_’ subunits. These trimmed and secreted subunits are further processed by the CylA serine protease to generate the active toxin subunits CylL_L_” and CylL_S_” [64,69]. The final gene in the cytolysin operon is *cylI*, which encodes the CylI immunity protein, a transmembrane protein of unknown function that confers self-protection to cytolysin-producing cells [66]. 

In the absence of target cells, CylL_L_” and CylL_S_” strongly associate to form inactive and insoluble multimeric complexes. However, when target cells are present the subunits interact, presumably to form a pore in the target cell membrane [71] (Figure 1C). The large subunit CylL_L_” has a greater affinity for the target cell membrane than the small subunit, which in the presence of a target cell is believed to result in a transient accumulation of excess free CylL_S_”, generating a quorum sensing autoinduction signal that triggers release of CylR2 and high level expression of the cytolysin operon. The CylL_S_” induction signal is believed to be transmitted in some way via the cell surface protein CylR1 [67].

### 3.2. Cytolysin Structural and Molecular Properties

The *E. faecalis* cytolysin components CylL_L_ and CylL_S_ have been classified as Type-A, pore-forming lantibiotics [72], and more recently as two-component, Class II lantibiotics [70]. Lantibiotics are complex polycyclic antimicrobial peptides, which are ribosomally synthesized by Gram-positive bacteria and are characterized by the presence of lanthionine and methyllanthionine bridges between dehydrated serine and threonine residues and cysteine thiols. Lantibiotics have extremely varied structures and functions, but they are all characterized by undergoing extensive post-translational modification and possessing either antibiotic or mophogenic activities [70]. Cytolysin appears to be unique among lantibiotics, in that it can lyse other bacteria as well as erythrocytes and other eukayotic cells [73]. The cytolysin subunits possess stretches of identity within the primary translation products, which likely target them through the same maturation pathways. They also show limited identity, but different bridging patterns, to beta-peptides (also called LanA2 peptides) of the two-component lantibiotics lacticin 3147 from *Lactococcus lactis* [74], and haloduracin from *Bacillus halodurans* [75]. 

**Figure 2:**
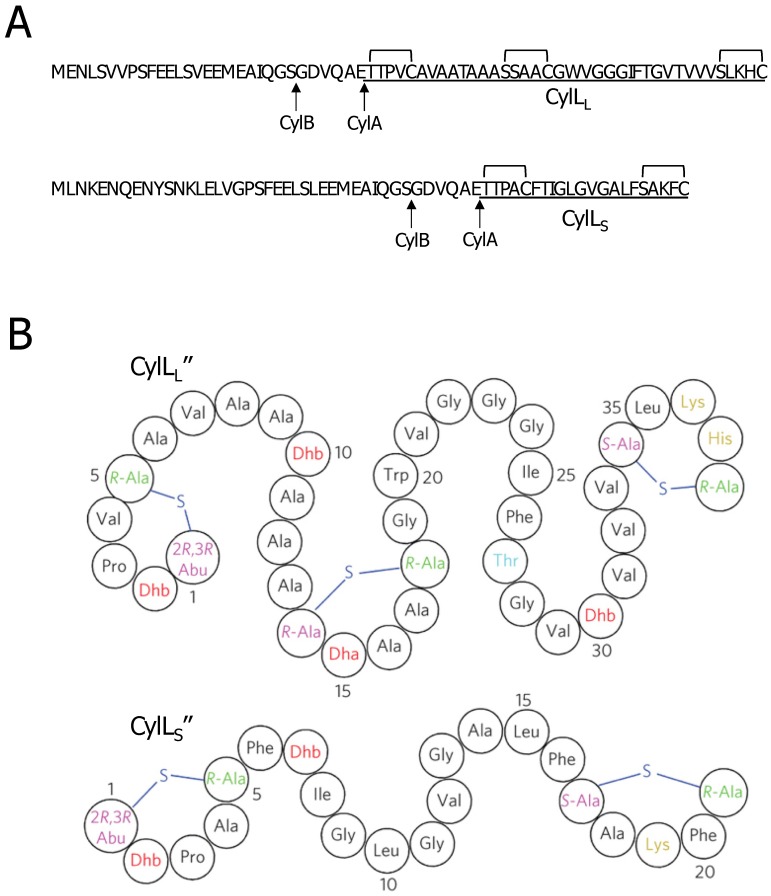
**Sequences and structures of the *E. faecalis* cytolysin subunits.** (**A**) Primary amino acid sequences of the cytolysin subunits CylL_L_ and CylL_S_. Arrows indicate sites of proteolytic cleavage by CylB and CylA [69], and brackets show the positions of lanthionine and methyllanthionine bridges. (**B**) Structures of the processed mature cytolysin subunits. Image is reproduced with permission from [76].

The positions of lanthionine linkages within the CylL_L_” and CylL_S_” peptides have recently been established [76] (Figure 2). Following ribosomal synthesis, the cytolysin subunit prepropeptides of 63 (CylL_S_) and 68 (CylL_L_) amino acids are modified post-translationally in the cytoplasm through reactions which are inferred to be catalyzed by CylM [65,72]. First, dehydration yields 2,3-didehydroalanine (Dha) from serine, and (*Z*)-2,3-didehydrobutyrine (Dhb) from threonine [70]. Then, neighboring intrapeptide cysteine residues make a nucleophilic, Michael addition to the dehydrated side chains, resulting in thioether bonds between the Dha (or Dhb) and cysteine side chains, creating the unusual amino acids lanthionine (when serine is the precursor) or methyllanthionine (when threonine is the precursor). Interestingly, the mature cytolysin peptides appear to adopt a unique stereochemistry, with CylL_L_” containing two lanthionine bridges in the unusual LL configuration, and CylL_S_” containing one [76]. Virtually all previously characterized lantibiotics contain bridges in the DL configuration. The functional consequence of this stereochemistry is currently unknown. The three-dimensional structures of the cytolysin peptides are also currently unknown, but recent advances in heterologous production of the subunits in *E. coli* will likely facilitate their determination [76].

Following modification of the cytolysin prepropeptides, the CylL_L_^*^ and CylL_S_^*^ propeptides are secreted from the cell by the product of the *cylB* gene [41]. During secretion, CylB removes 24 amino acids from the amino terminus of CylL_L_^*^ and 36 amino acids from the amino terminus of CylL_S_^*^. This removal is believed to be catalyzed by a cysteine protease domain within CylB [41], and cleavage occurs within a nearly identical stretch of 26 amino acids in the otherwise structurally dissimilar subunits [69]. Whether all or part of these conserved 26 amino acid leader sequences constitutes a trafficking signal for CylM-mediated modification or CylB secretion remains to be explored.

Precisely how the cytolysin modification enzymes CylM, CylB, and CylA are produced, processed and sent to their final destinations is unclear. The *E. coli* hemolysin A toxin (HlyA) is processed and secreted by a type 1 secretion system consisting of the inner membrane protein HlyB, the membrane fusion protein HlyD, and the outer membrane protein TolC, which form a continuous but transient translocator from the cytosol directly out of the cell to allow for HlyA secretion [77].Complex natural products, including antibiotics, are also synthesized in processive steps by multienzyme megasynthase complexes as large as 2 MDa [78]. Experimental evidence suggests that the proteins involved in post-translational modification and secretion of the lantibiotic subtilin might also organize into a membrane bound complex [79]. Because of the need for processivity in the maturation of the cytolysin prepropeptides [65], it seems possible that CylM, CylB, and CylA may similarly be organized in a transmembrane complex that efficiently modifies, secretes and activates each subunit, but this remains to be shown. 

### 3.3. Cytolysin Regulation

The cytolysin operon contains two promoters; the P_L_ promoter regulates transcription of genes related to toxin structure and function (*cylL_L_*, *cylL_S_*, *cylM*, *cylB*, *cylA*, and *cylI*), while the P_Reg_ (P_R_) promoter overlaps with P_L_ and regulates transcription of the regulatory genes *cylR1* and *cylR2*, which are transcribed in the opposite direction from the rest of the operon [67,68] (Figure 1A). In the uninduced state, the cytolysin operon is believed to be transcribed at a low level, so that a small amount of all system components are available to respond to the presence of target cells when the need arises [67]. When target cells are present, the large subunit CylL_L_” preferentially binds to cell membranes with greater affinity than the small subunit CylL_S_”, leading to a transient accumulation of free small subunit in solution [71]. Once the concentration of free CylL_S_” exceeds a threshold, it induces transcription of the cytolysin operon from the P_L_ promoter, presumably through altered association or dissociation of the CylR2 protein from the promoter region. The crystal structure of CylR2 was solved and the precise nature of its binding to P_L_ has been determined *in vitro* [68]. It is also known that CylR1, a suspected membrane protein, is required for induction of the cytolysin operon [67]. However, the precise mechanism of how the accumulation of extracellular CylL_S_” is transmitted to intracellular CylR2, as well as the role of CylR1 in transmitting this signal, are not currently understood. 

While it appears that the genes within the cytolysin operon are transcribed polycistronically, and that the operon contains at least two promoters, there is some experimental evidence to suggest that transcription may be more complex. Based on the behavior of transposon insertion mutants, the final two genes within the cytolysin operon, *cylA* and *cylI*, were originally thought to be transcribed independently from the rest of the operon [64,66]. However, promoter elements besides P_L_ and P_R_ have yet to be identified. In the active state, transcripts from *cylL_L_* and *cylL_S_* are far more abundant than transcripts of any other cytolysin components, possibly due to a stem-loop structure between *cylL_L_* and *cylM* that may form a conditional terminator element [80]. Prior experiments that focused on quantifying transcription of the various cytolysin operon components have relied on PCR-based approaches [80], which can artificially simplify the picture through selective amplification of a preferred species. Newly developed technologies, such as RNA sequencing (RNA-seq), would allow more precise quantification of expression levels of all operon components simultaneously, and can distinguish the directionality of transcription as well as transcription initiation from processing sites [81]. 

As noted above, CylR1 plays a role in transmitting the induction signal or otherwise facilitates induction of cytolysin operon transcription in the presence of target cells [37,67], but the mechanism is not obvious. One possibility is that CylR1 and CylR2 may form a novel two-component regulatory system that lacks the phosphorelay elements common to classical bacterial two-component systems [82]. CylR1 contains three predicted alpha-helical transmembrane domains, and is therefore believed to localize to the cell membrane, but this awaits verification. As a membrane protein, CylR1 could sense excess CylL_S_”, either in the environment or in contact with the membrane. Previous models have depicted CylR1 associating directly with CylR2, suggesting that a conformational change initiated by CylR1 causes CylR2 to dissociate with the P_L_ promoter region [67,83]. Alternately, CylR1 could facilitate CylL_S_” internalization into the cytoplasm, perhaps in association with a cellular oligopeptide permease, similar to the mechanism of internalization in *E. faecalis* for pheromone signaling [84]. 

### 3.4. Toxin Mechanism of Action

Very little is currently known regarding the nature of the interaction between cytolysin toxin subunits, either in the presence or the absence of target cells. The large subunit CylL_L_” binds to target cells with about a seven-fold greater affinity than CylL_S_” [71]. Interestingly, the immediate precursors of the active toxin subunits, CylL_L_’ and CylL_S_’, are only six amino acids longer than the fully mature subunits, yet these precursors do not detectably associate with each other, and have no detectable hemolytic activity [69]. This suggests that the amino terminus of the fully processed toxin subunits is instrumental in their association with membranes and into polymers. 

Exactly how the CylL_L_” and CylL_S_” subunits compromise target cell membranes leading to lysis is unclear, but is likely to bear some similarity to pore formation by the well-studied lantibiotics nisin and lacticin 3147, both produced by *Lactococcus lactis* [85,86]. Nisin forms pores via a multi-step process involving: (1) binding to the bacterial cell wall precursor molecule lipid II; and (2) reorientation of nisin molecules from parallel to perpendicular to the membrane surface [87]. The amino-terminal rings of nisin bind to lipid II, and the carboxy-terminus interacts with the lipid bilayer of the target bacterial cell. Accumulation of lipid II and nisin in this way results in a pore formed by four lipid II and eight nisin molecules in an unknown structural arrangement [88]. Pore formation by lacticin 3147, a two-component lantibiotic, also involves multiple steps: (1) the LtnA1 subunit first associates with the membrane; (2) it forms a complex with the LtnA2 subunit in a 1:1 stoichiometry; and (3) LtnA2 in the complex then enters the membrane and forms a pore [89]. Whether the cytolysin subunits interact in a similar way, as well as their stoichiometry, remains to be determined.

Target cell surface receptor, or cell surface receptors, that enable cytolysin-mediated lysis are unknown. Cytolysin is unique among lantibiotics in its ability to lyse a broad range of cells, including bacteria, various mammalian erythrocytes, and other eukaryotic cells [90]. If there is a specific receptor, it would have to be highly conserved across widely divergent kingdoms. As noted above, nisin and many other lantibiotics use lipid II as a docking molecule [91,92]. This could be a possible candidate for cytolysin targeting of bacteria, but this would invoke different mechanisms for prokaryotic and eukaryotic cell lysis. A higher membrane phosphatidylcholine content has been found in those erythrocytes that are most susceptible to lysis by cytolysin [34], and both sphingomyelin and phosphatidylcholine inhibit the lysis of horse erythrocytes by cytolysin [44]. Finally, cytolysin activity could be due to general membrane properties, with susceptibility at least in part attributable to the absence of an inhibitor on the target cell surface, such as lecithin [39]. 

The way in which cytolysin-producing cells are protected from self-lysis, and how immunity is transferred between cells, also are not well understood. Other lantibiotic-producing bacteria are protected from self-lysis by immunity proteins and/or ABC transporters that serve to decrease the local concentration of the lantibiotic [93,94]. In *E. faecalis*, the immunity factor CylI, an apparent transmembrane protein with possible zinc metalloprotease activity, was shown to be necessary and sufficient to confer protection from cytolysin-mediated bacterial cell death [66]. It is unknown whether CylI interacts with and/or cleaves one or both cytolysin subunits, but it seems possible that CylI could prevent pore formation by cleaving subunits that attempt to embed within the producer cell membrane, or by cleaving proteinaceous target cell receptors, should they exist. Because the cytolysin operon is encoded on transmissible plasmids and a mobile pathogenicity island, an important unanswered question remains as to how the operon encoding the cytolysin is transferred from an immune-producing cell to a susceptible recipient without first killing the recipient. One possible explanation for recipient cell protection might be the need for high bacterial cell levels to induce cytolysin expression [67]. Perhaps the pheromone quorum signaling pathway involved in pAD1 transfer is triggered at lower cell densities, before the threshold for derepression of cytolysin expression is reached.

### 3.5. Biological Role of Cytolysin

Many studies of the *E. faecalis* cytolysin are motivated by findings that this molecule exacerbates infection in humans and model systems [22,43,51]. However, because of the relative rarity of *E. faecalis* infection in comparison to its abundance as a commensal in the GI tract of diverse animals, it seems likely that this toxin evolved for a more common purpose, where positive selection is more likely to apply. For a commensal microbe that is dependent upon its host (and the microbial community that the host supports) to fulfill its auxotrophies, it seems probable that selection for the cytolysin occurred in an environment that was mutually beneficial to both *E. faecalis* and its host. Possible cytolysin activities that could benefit a host might include: providing a defense against something that is more harmful (such as an intestinal parasite), acting as a colonization factor, or facilitating nutrient acquisition from prokaryotic or eukaryotic sources. Perhaps the bacteriocin activity of the cytolysin allows *E. faecalis* to occupy a novel host niche that non-cytolytic bacteria cannot access. The impact that cytolysin production has on the host microbiome has not yet been investigated, although recent advances in microbial ecology and metagenomics should be able to readily address this question in humans [95], or other natural hosts [96,97]. Additionally, *E. faecalis* can incorporate exogenous hemin into its cytochromes, and this was found to provide a growth advantage under aerobic conditions [11,98]. Perhaps the ability to co-opt extracellular hemin from a host or neighboring organism confers a large enough growth advantage to drive the evolution of target cell lysis by cytolysin. 

In addition to possible roles in colonization and nutrient acquisition, the cytolysin appears to function at least in part as a signaling molecule that can monitor bacterial population size and probe the environment for target cells [83,99]. Cytolysin subunits are produced and secreted into the environment, but their relative abundance is also monitored by the producer cell and when target cells are close by, the small subunit CylL_S_” becomes a signaling molecule that induces a change in gene expression, turning on production of additional cytolysin subunits [71]. The ability to recognize the presence or absence of target cells allows *E. faecalis* to respond to its environment in a more nuanced way, and may contribute to the successful colonization of many different environmental niches.

## 4. Conclusions

The recent evolution of *E. faecalis* strains that are both hypervirulent and multidrug resistant underscores the need for a better understanding of the biology of this important pathogen. The cytolysin forms a critical part of this understanding, as it contributes more to infection toxicity than any other *E. faecalis* factor studied, and it likely also allows *E. faecalis* to colonize new ecologies. A better understanding of the *E. faecalis* cytolysin may aid in understanding the biological mechanisms of other lantibiotics, as well as deepen our knowledge of how *Enterococcus* evolved this molecule in the first place. Application of the latest genomics-age technologies will certainly shed new light on the biology of the *E. faecalis* cytolysin, and will provide a more complete understanding of the structure and function of this important molecule.
